# Intracerebral Hematoma Secondary to Wernicke’s Encephalopathy: A Case Report With a Review of Literature

**DOI:** 10.7759/cureus.78330

**Published:** 2025-02-01

**Authors:** Khadija Ouchen, Siham Bouchal, Nizar El Bouardi, Mustapha Maaroufi, Faouzi Belahsen

**Affiliations:** 1 Neurology, Hassan II University Hospital, Fez, MAR; 2 Laboratory of Epidemiology, Clinical Research, and Health Community, Faculty of Medicine and Pharmacy, Sidi Mohamed Ben Abdellah University, Fez, MAR; 3 Radiology, Hassan II University Hospital, Fez, MAR

**Keywords:** cortical ribbon, incoercible vomiting, intracranial hemorrhage, thiamin deficiency, wernicke's encephalopathy

## Abstract

Wernicke’s encephalopathy (WE) is an acute neurological disorder secondary to thiamine deficiency (vitamin B1). Treatment should be started as early as possible to prevent serious complications. Typical magnetic resonance imaging (MRI) findings of WE can be seen as symmetrical signal abnormalities in the periventricular regions of the thalamus, hypothalamus, mammillary bodies, periaqueductal region, and floor of the fourth ventricle. Intracranial hemorrhage is a rare but serious complication of WE, whose physiopathology remains unclear. We report the case of a 48-year-old man with suggestive MRI abnormalities of WE in typical sites and low serum thiamine levels, complicated by intracerebral hematoma.

## Introduction

Wernicke’s encephalopathy (WE) is an acute neuropsychiatric condition secondary to thiamine deficiency, characterized by a clinical triad of ophthalmoplegia, ataxia, and altered mental status [1-3]. The diagnosis can sometimes be challenging due to nonspecific clinical signs, leading to underdiagnosis [2]. Early treatment by parental thiamine is essential, as WE is associated with high rates of morbidity and mortality [1,3]. Magnetic resonance imaging (MRI) often shows classic abnormalities suggestive of WE [2,3]. The mortality rate of WE remains at 10%-20% due to delayed diagnosis [1]. We report a rare case of intracranial hemorrhage related to WE in a 48-year-old male patient with a history of incoercible vomiting, with a literature review of similar cases.

## Case presentation

A 48-year-old man was admitted to our emergency department (ED) in a state of coma with respiratory distress. He has a history of cannabis-alcohol exposition and a medical history of toxic stable polyneuropathy for two years. The patient has no significant disability and can carry out all activities, with a modified Rankin score of 1. Two weeks before his admission, he experienced epigastric pain and incoercible vomiting that did not improve with medications. Three days before admission, he developed acute confusion, diplopia, gait instability, and visual hallucinations. He initially underwent investigations in a private clinic, and without a diagnosis, the patient was transferred to our ED. On admission, the patient was unconscious, with a Glasgow Coma Scale score of 8, in respiratory distress, and afebrile with normal blood pressure and normal blood sugar level. He required intubation and sedation immediately.

**Figure 1:**
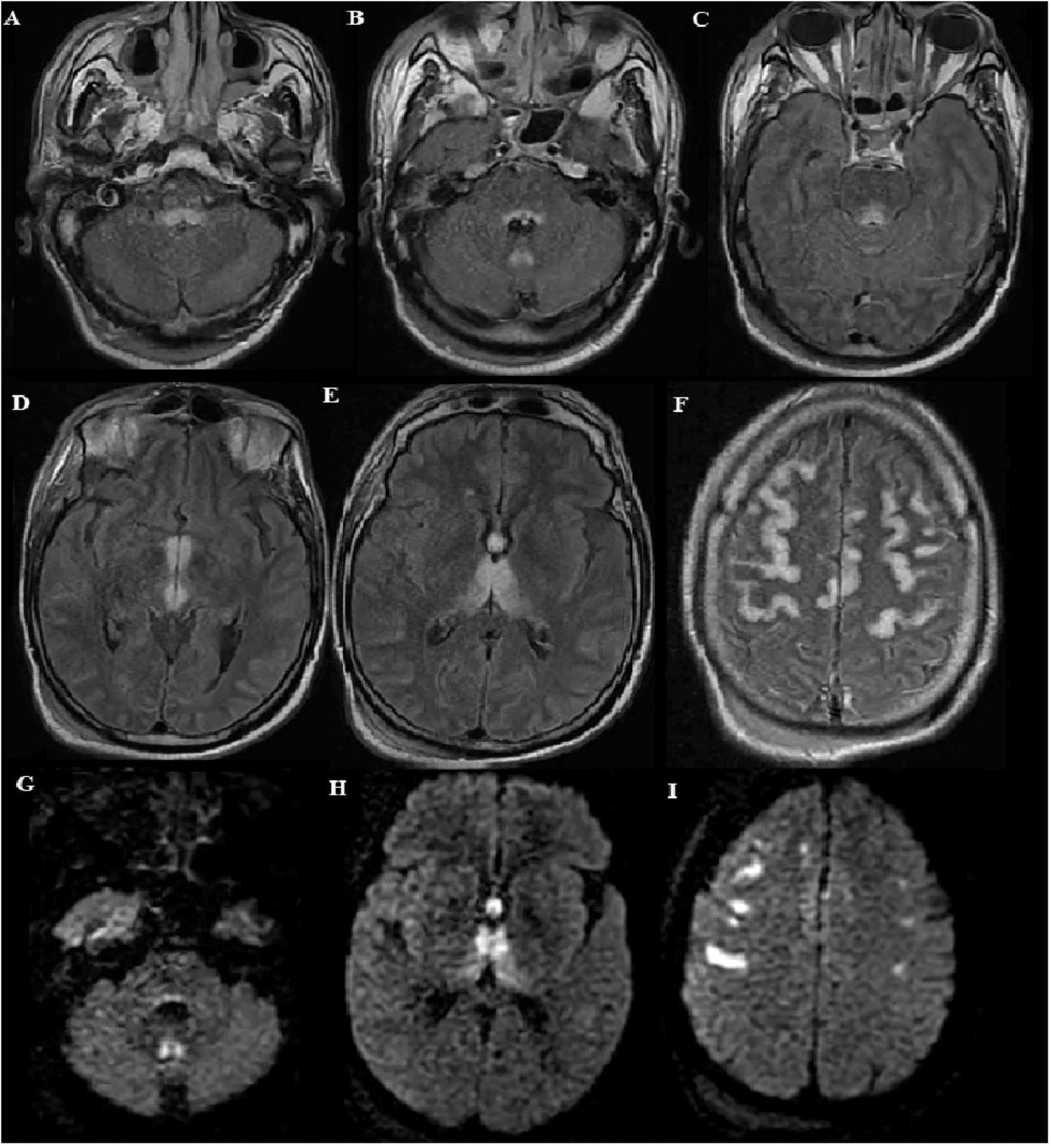
(A-I) FLAIR images (A-F) and diffusion weighted image (DWI) (G-I) of the brain: high signal lesions were seen in the bilateral mammillary bodies, tectal plate, periaqueductal gray matter of the midbrain, posterior pons, bilateral medial thalamic nuclei , midline cerebellum , and the cortical frontal ribbon FLAIR: fluid-attenuated inversion recovery

Brain MRI revealed symmetric hyperintensities on fluid-attenuated inversion recovery (FLAIR), involving bilateral medial thalamic nuclei, bilateral mammillary bodies, periaqueductal gray matter of the midbrain, tectal plate, posterior pons, and midline cerebellum. Additionally, there were bilateral hyperintensities FLAIR lesions along the cortical frontal ribbon. No microbleeds were observed on T2* sequences, and there was no contrast enhancement on T1 post-gadolinium sequences (Figure 1).

**Figure 2:**
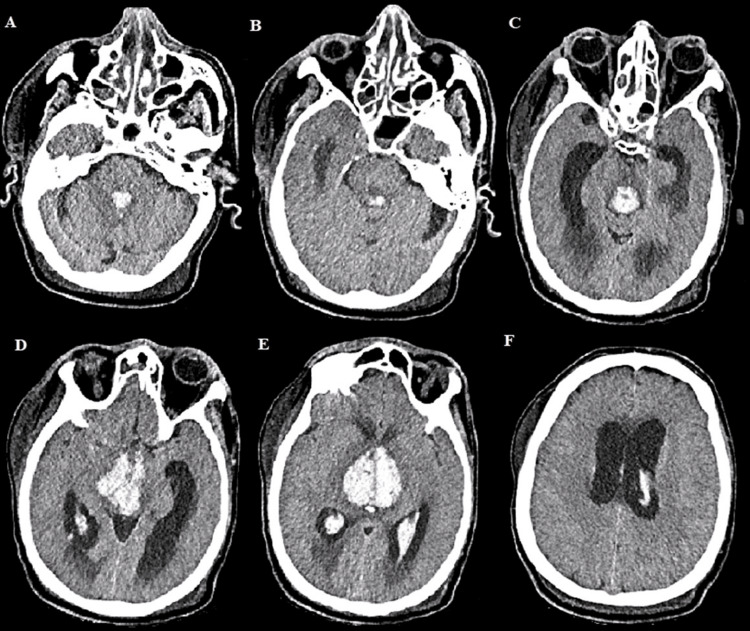
(A-F) Cerebral CT scan showed a bithalamic, periaqueductal and intraventricular hemorrhage, with acute hydrocephalus

The cerebrospinal fluid was normal and serum assessment revealed acute functional renal failure. The clinical presentation and imaging findings were suggestive of WE. He was promptly treated intravenously with 500 mg of vitamin B1 (thiamine) for five days and supportive care (assisted ventilation, intravenous rehydration). Serum assessment revealed a low thiamine level of 11 μg/L (normal range 28-85 μg/L), and the diagnosis of WE has been confirmed. Four days later, he developed right anisocoria, followed by bilateral mydriasis, and hemodynamic instability. A brain CT scan showed bithalamic hemorrhages as well as bleeding in the periaqueductal gray matter and intraventricular hemorrhage involving the bilateral lateral ventricles, third ventricle, and fourth ventricle, with acute hydrocephalus (Figure 2). The patient died after a few days in intensive care.

## Discussion

Thiamine is a primordial cofactor in glucose oxidation by neurons due to its role. It is necessary for the intermediate metabolism of carbohydrates and lipids, and the production of amino acids and neurotransmitters derived from glucose [2,3]. It also potentiates the acetylcholine’s biological effect [3]. Humans need a daily dose of thiamine of 1 to 2 mg. The body’s reserves of this vitamin are rapidly depleted in prolonged malnutrition [3]. Thiamine deficiency leads, in the acute phase, to vascular congestion, microglial proliferation, and petechial hemorrhages, with lactate production leading to acidosis and cytotoxic edema [2]. In the chronic phase, demyelination occurs with neuronal loss and gliosis. Atrophy of the mammillary bodies is quite common in cases progressing to Korsakoff syndrome. The reasons for the selective vulnerability of specific brain regions to thiamine deficiency are still not fully understood [4,5].

WE is a neurological disorder secondary to thiamine deficiency, a common condition in chronic alcoholism. There are other causes of WE which were reported in the literature, including hyperemesis gravidarum, chronic diarrhea, prolonged fasting, hemodialysis, hyperuremia, gastric surgery, and cancer and chemotherapeutic treatments [3]. The classical triad of clinical symptoms includes acute altered state of consciousness ranging from stupor to coma, oculomotor abnormalities, and cerebellar or vestibular ataxia [3]. WE is often underdiagnosed since only 16%-33% of patients carry all features of the clinical triad [1,2,4].

MRI is currently considered as the reference imaging device to confirm the diagnosis of WE. It classically shows symmetric hyperintensities on T2/FLAIR in the thalami, tectal plate, mammillary bodies, and periaqueductal [2,3]. Additionally, MRI findings can be seen in the cerebellar vermis, the cranial nerve nuclei, the dentate nucleus, the caudate nucleus, the red nucleus, the splenium of the corpus callosum, and the cerebral cortex [6,7]. Cortical involvement reflects the severity of WE and is associated with a poor prognosis. It frequently affects the frontal and parietal lobes, particularly around the central sulcus [8-9]. The lesions as mentioned earlier can show abnormal signals on diffusion-weighted imaging (DWI), with or without contrast enhancement. The cerebral MRI has a sensitivity of only 53% but has a high specificity of 93% [3]. The absence of these MRI abnormalities should not exclude the diagnosis if the clinical context is evident [4]. 

Intracranial hemorrhage is a rare but serious complication of WE and its mechanism remains mysterious. In a cohort published in 1965 involving 43 patients with WE, 60% of patients had intracranial hemorrhages, of which only 16% were macroscopically identifiable. The authors proposed two factors explaining the hemorrhagic complications. The first factor is a generalized hemorrhagic diathesis, often associated with hepatic insufficiency, thrombocytopenia, or the administration of thromboprophylaxis. The second factor, related to the thiamine deficiency itself, involves the localization and severity of the lesions, alongside the presence of microscopic petechial hemorrhages. Hemorrhagic complications are precipitated by renal or hepatic failure, which is common in these patients, though not always present [5].

Thiamine deficiency leads to cytotoxic edema in specific areas of the central nervous system, followed by hemorrhage due to vasodilation. The hemorrhage can occur without parenchymal changes, as it is not solely secondary to necrosis but also results from hemodynamic changes caused by vascular dysfunction mediated by vitamin B1 deficiency [10].

Table 1 summarizes the literature cases of WE with intracranial hemorrhagic complications. Malnutrition is the common etiology of WE. Three patients had acute renal failure, four patients had thrombocytopenia, one patient had liver failure, and one other patient had a mild elevation of transaminases, which confirmed the hypothesis of Rosenblum WI about the presence of a generalized hemorrhagic diathesis [5]. Only one patient was given anticoagulants. The common localizations of bleeding are thalami, ventricles, and mammillary bodies. There are some cases with a good prognosis. 

**Table 1:** Summary of literature’s cases of Wernicke’s Encephalopathy with intracranial hemorrhage M: male; F: female; INR: international normalized ratio

Reference	Age (years)/Sex	Symptoms	Time to diagnosis and supplementation	Serum thiamine levels	Other factors	Cause of WE	Localization of bleeding	Outcome
Pfister et al. [11]	58 F	Acute confusional state, with nystagmus and ataxia	Necropsy (no supplementation)	Not done	INR 1.37	Acute vomiting due to candidiasis of the esophagus	Intraventricular	Poor (death)
Susuki et al. [12]	74 F	Acute confusional state with gait disturbance (administration of intravenous heparin for subendocardial ischemia)	6 days	<5ng/mL	Activated thromboplastin time 45.9	Malnutrition	Mammillary bodies, medial nuclei of the thalamus, superior and inferior colliculi, right precentral gyrus, subarachnoid spaces, intraventricular	Poor (severe sequelae)
Helbok et al. [13]	29 F	Altered mental status, tetraparesis with gaze deviation	Unknown	Unknown	Acute renal failure	Malnutrition	Paramedian thalamic nuclei, periaqueductal Intraventricular	Good (partial recovery)
Shin et al. [14]	40 F	Gradual confusional state, with ophthalmoplegia and nystagmus	Unknown	Unknown	Unknown	Unknown	Bilateral inferior colliculi	Good (partial recovery)
Pereira et al. [15]	27 F	Acute confusional state, with tetraparesis and cranial nerves palsy	2 weeks	Unknown	Unknown	Malnutrition due to colectomy	Bilateral frontal cortical lesions	Good (partial recovery)
Busani et al. [16]	46 F	Acute agitated state	7 days	Unknown	Thrombocytopenia (34000/μl), renal failure, liver failure	Malnutrition	Mammillary bodies, quadrigeminal plate	Poor (death due to a septic shock)
Kim and Kim [17]	34 M	Acute confusional state, with ophthalmoplegia and ataxia	2 weeks	Unknown	Unknown	Malnutrition due to diarrhea	Bilateral midbrain tectum, medial caudal thalamus	Poor (death due to a septic shock)
Jeon et al. [4]	56 M	Altered mental state	3 weeks	Unknown	Thrombocytopenia (18000 μl-1)	Malnutrition	Thalamus and periaqueductal gray matter and intraventricular	Unknown
Al-Bayati et al. [18]	48 M	Acute confusional state	6 weeks	37 μg/dL (normal range 78–185 μg/dL)	Thrombocytopenia (71 000 per mm3) Slight elevation of INR Acute renal failure Mild elevation of transaminases	Malnutrition	Bilateral thalamic third ventricle	Good (partial recovery)
DePolo et al. [19]	31 M	Altered mental status with gait instability and complete ophthalmoplegia	Several weeks	24 nmol/L (normal range 78-185)	thrombocytopenia (platelet count 99 k -> 54 k/uL on hospital day 2)	Malnutrition	Thalamus and intraventricular	Good (partial recovery)
Tokue et al. [20]	60 F	Altered consciousness	Unknown	6 ng/mL (normal range 28-56 ng/mL)	No abnormalities	Malnutrition associated with depression	Thalamus	Poor (severe sequelae)
Our case	48 M	Acute confusional state with diplopia, and visual hallucinations, then a coma	10 days	11 μg/L (normal range 28-85 μg/L)	Acute renal failure	Acute incoercible vomiting	Thalami periaqueductal gray matter intraventricular	Poor (death)

## Conclusions

WE is a medical emergency and is considered a reversible condition; severe neurological sequelae including Korsakoff syndrome and death might happen if this condition is untreated or treated late. Our case highlights a rather rare complication of WE, which is an intracranial hemorrhage that has been reported in the literature. This may be due to the microscopic petechial hemorrhages in areas affected by thiamine deficiency, which can be precipitated by other coexisting factors (thrombocytopenia, hepatic or renal insufficiency, thromboprophylaxis). However, the exact pathophysiology remains unclear and needs to be elucidated by further studies.

## References

[1] Thomson AD, Cook CC, Guerrini I, Sheedy D, Harper C, Marshall EJ (2008). Wernicke's encephalopathy revisited. Translation of the case history section of the original manuscript by Carl Wernicke 'Lehrbuch der Gehirnkrankheiten fur Aerzte and Studirende' (1881) with a commentary. Alcohol Alcohol.

[2] Sechi G, Serra A (2007). Wernicke's encephalopathy: new clinical settings and recent advances in diagnosis and management. Lancet Neurol.

[3] Bouchal S, Bougtoub N, Alami B, Chtaou N, Maaroufi M, Belahsen F (2020). Gayet-Wernicke encephalopathy: clinical features and radiological anomalies (Article in French). Pan Afr Med J.

[4] Jeon S, Kang H (2016). Wernicke's encephalopathy with intracranial hemorrhage. iMRI.

[5] Rosenblum WI, Feigin I (1965). The hemorrhagic component of Wernicke's encephalopathy. Arch Neurol.

[6] Zuccoli G, Pipitone N (2009). Neuroimaging findings in acute Wernicke's encephalopathy: review of the literature. AJR Am J Roentgenol.

[7] Zuccoli G, Santa Cruz D, Bertolini M, Rovira A, Gallucci M, Carollo C, Pipitone N (2009). MR imaging findings in 56 patients with Wernicke encephalopathy: nonalcoholics may differ from alcoholics. AJNR Am J Neuroradiol.

[8] Ota Y, Capizzano AA, Moritani T, Naganawa S, Kurokawa R, Srinivasan A (2020). Comprehensive review of Wernicke encephalopathy: pathophysiology, clinical symptoms and imaging findings. Jpn J Radiol.

[9] Sakurai K, Sasaki S, Hara M, Yamawaki T, Shibamoto Y (2009). Wernicke's encephalopathy with cortical abnormalities: clinicoradiological features: report of 3 new cases and review of the literature. Eur Neurol.

[10] Chen Q, Okada S, Okeda R (1997). Causality of parenchymal and vascular changes in rats with experimental thiamine deficiency encephalopathy. Pathol Int.

[11] Pfister HW, von Rosen F, Bise K (1995). Severe intraventricular haemorrhage shown by computed tomography as an unusual manifestation of Wernicke's encephalopathy. J Neurol Neurosurg Psychiatry.

[12] Susuki K, Ueda N, Tanigawa A, Horikoshi H, Kuroiwa Y (2002). Wernicke's encephalopathy accompanied by multiple symptomatic cerebral hemorrhages during the recovery phase. Eur Neurol.

[13] Helbok R, Beer R, Engelhardt K (2008). Intracerebral haemorrhage in a malnourished patient, related to Wernicke's encephalopathy. Eur J Neurol.

[14] Shin NY, Nam HS, Lee SK (2009). Hemorrhagic Wernicke encephalopathy in a patient with liver transplantation. Neurology.

[15] Pereira DB, Pereira ML, Gasparetto EL (2011). Nonalcoholic Wernicke encephalopathy with extensive cortical involvement: cortical laminar necrosis and hemorrhage demonstrated with susceptibility-weighted MR phase images. AJNR Am J Neuroradiol.

[16] Busani S, Bonvecchio C, Gaspari A, Malagoli M, Todeschini A, Cautero N, Girardis M (2014). Wernicke's encephalopathy in a malnourished surgical patient: a difficult diagnosis. BMC Res Notes.

[17] Kim TW, Kim JS (2014). Bilateral midbrain and thalamic hemorrhage in Wernicke encephalopathy. Can J Neurol Sci.

[18] Al-Bayati AR, Nichols J, Jovin TG, Jadhav AP (2016). Thiamine deficiency presenting as intraventricular hemorrhage. Stroke.

[19] DePolo D, Gillen S, Marden K, Rajagopalan S, Thon OR, Siegler JE, Thon JM (2022). Severe Wernicke's encephalopathy associated with cortical ribboning and intracranial hemorrhage. Neurohospitalist.

[20] Tokue H, Ishikawa R, Oshima K (2024). Wernicke encephalopathy with atypical imaging findings in a depressed patient: a case report. Radiol Case Rep.

